# Ventilation Induces Changes in Pulse Wave Transit Time in the Pulmonary Artery

**DOI:** 10.3390/biomedicines11010182

**Published:** 2023-01-11

**Authors:** Fabian Mueller-Graf, Paul Frenkel, Chiara Felicitas Albus, Maike Henkel, Susanne Reuter, Brigitte Vollmar, Gerardo Tusman, Andy Adler, Sven Pulletz, Stephan H. Böhm, Amelie Zitzmann, Daniel A. Reuter

**Affiliations:** 1 Department of Anesthesiology, Intensive Care Medicine and Pain Therapy, University Medical Center Rostock, Schillingallee 35, 18057 Rostock, Germany; 2 Rudolf-Zenker-Institute for Experimental Surgery, University Medical Center Rostock, 18057 Rostock, Germany; 3 Department of Anesthesiology, Hospital Privado de Comunidad, Mar de Plata 7600, Argentina; 4 Systems and Computer Engineering, Carleton University, Ottawa, ON K1S5B6, Canada

**Keywords:** pulmonary artery pressure (PAP), pulmonary hypertension (PH), pulse wave transit time (PWTT), heart lung interaction

## Abstract

Pulse wave transit time (PWTT) shortens as pulmonary artery pressure (PAP) increases and was therefore suggested as a surrogate parameter for PAP. The aim of this analysis was to reveal patterns and potential mechanisms of ventilation-induced periodic changes in PWTT under resting conditions. To measure both PWTT and PAP in five healthy pigs, two pulmonary artery Mikro-Tip™ catheters were inserted into the pulmonary vasculature: one with the tip placed in the pulmonary artery trunk, and a second one placed in a distal segment of the pulmonary artery. Animals received pressure-controlled mechanical ventilation. Ventilation-dependent changes were seen in both variables, PWTT and mean PAP; however, changes in PWTT were not synchronous with changes in PAP. Thus, plotting the value of PWTT for each heartbeat over the respective PAP revealed a characteristic hysteresis. At the beginning of inspiration, PAP rose while PWTT remained constant. During further inspiration, PWTT started to decrease rapidly as mPAP was about to reach its plateau. The same time course was observed during expiration: while mPAP approached its minimum, PWTT increased rapidly. During apnea this hysteresis disappeared. Thus, non-synchronous ventilation-induced changes in PWTT and PAP were found with inspiration causing a significant shortening of PWTT. Therefore, it is suggested that the respiratory cycle should be considered when using PWTT as a surrogate for PAP.

## 1. Introduction

Pulmonary hypertension (PH) is known to lead to right and global heart failure, and is associated with a high one-year mortality [1]. In current guidelines, pulmonary hypertension (PH) is defined as a mean pulmonary artery pressure (mPAP) above 20 mmHg measured by invasive right heart catheterization at rest [1]. Unfortunately, current non-invasive methods such as echocardiography or magnetic resonance imaging (MRI) are not accurate enough to confirm the diagnosis of PH or to serve as follow-up [2,3,4]. Therefore, the determination of pulse wave transit time (PWTT) has been proposed as a non-invasive surrogate for pulmonary artery pressure (PAP) [5].

In a previous publication, we described a strong negative linear correlation between the PWTT and sPAP, mPAP and dPAP over a wide range of pressures in a porcine model of acute PH [6]. Besides the expected rather large PAP-induced changes in PWTT we also noticed a range of PWTT values (10 to 15 ms) for one corresponding PAP value, which resulted in a blurring of the dot plots of PWTT over PAP. A more detailed analysis revealed a periodic ventilation-dependent variation of PWTT of the heartbeats. Therefore, we decided to investigate these ventilation-induced effects in more detail starting with a review of the literature.

No publication investigating the direct effects of ventilation on PWTT in the lung could be found. Nevertheless, some publications concerned themselves with diseases of the respiratory system and their effects on PWTT or pulse wave velocity (PWV), defined as the PWTT divided by the measured length in the pulmonary artery. Recently, in patients suffering from chronic obstructive pulmonary disease (COPD), longer PWTTs than those of healthy subjects were reported using MRI and transthoracic echocardiography together [7]. This was explained by a loss of arterial stiffness due to endothelial dysfunction and destruction. An analysis of patients with pulmonary hypertension due to pulmonary fibrosis revealed a shorter PWTT compared to healthy individuals [8]. The above-mentioned studies, however, did not address the potential effect that breathing might have on PWTT. Since our data revealed evidence for a dependency of PWTT on the state of the respiratory cycle we decided to analyze this phenomenon in more detail and to relate these to the pulmonary artery pressures.

Based on the finding of our previous study, we hypothesized that a ventilation-dependent relationship between PWTT and PAP exists [6]. Thus, the goal of this analysis of our previously published animal study was to reveal patterns and potential mechanisms of ventilation-induced periodic changes in PWTT under resting conditions.

## 2. Materials and Methods

### 2.1. Animal Model and Anesthesia

The study was approved by the governmental ethical board for animal research (Landesamt für Landwirtschaft, Lebensmittelsicherheit und Fischerei, Mecklenburg-Vorpommern, Germany; No: 7221.3-1-037/19) and was carried out in accordance with the EU-directive 2010/63/EU and the Animal Research: Reporting of In Vivo Experiments guidelines 2.0 (ARRIVE 2.0) [9]. The data obtained under resting conditions of the animals presented here have neither been analyzed nor published before except those data registered during experimentally induced pulmonary hypertension [6]. Of the 6 animals studied, the data of five healthy German Landrace pigs (24.4–48.3 kg, 12–15 weeks old) were of sufficient quality to be analyzed. Detailed information about the animal model and anesthesia can be found in the above-mentioned previous publication [6].

### 2.2. Ventilation

The pigs were intubated endotracheally (ID 7.0 mm) and mechanically ventilated in a pressure-controlled mode using a Dräger Primus ventilator (Dräger Medical, Lübeck, Germany) with tidal volumes of 6 mL/kg. The respiratory rate was adjusted to maintain end-tidal partial pressure of CO_2_ at 5 ± 0.4 kPa. Airway pressure (Paw) was measured at the heat moisture exchange filter using a disposable pressure sensor with a PowerLab 16/35 (ADInstruments, Dunedin, New Zealand). Esophageal pressure (Peso) was measured using the same disposable pressure sensor connected to a NutriVent multifunctional nasogastric catheter (Sidam Group, San Glacomo Roncole, Italy) positioned according to Baydur [10]. PEEP was individually adjusted between 5 and 8 mbar to obtain a transpulmonary pressure just above zero as recommended by Talmor, where transpulmonary pressure (Ptrans) was calculated as the difference between Paw and Peso [11].

### 2.3. Instrumentation

**Figure 1:**
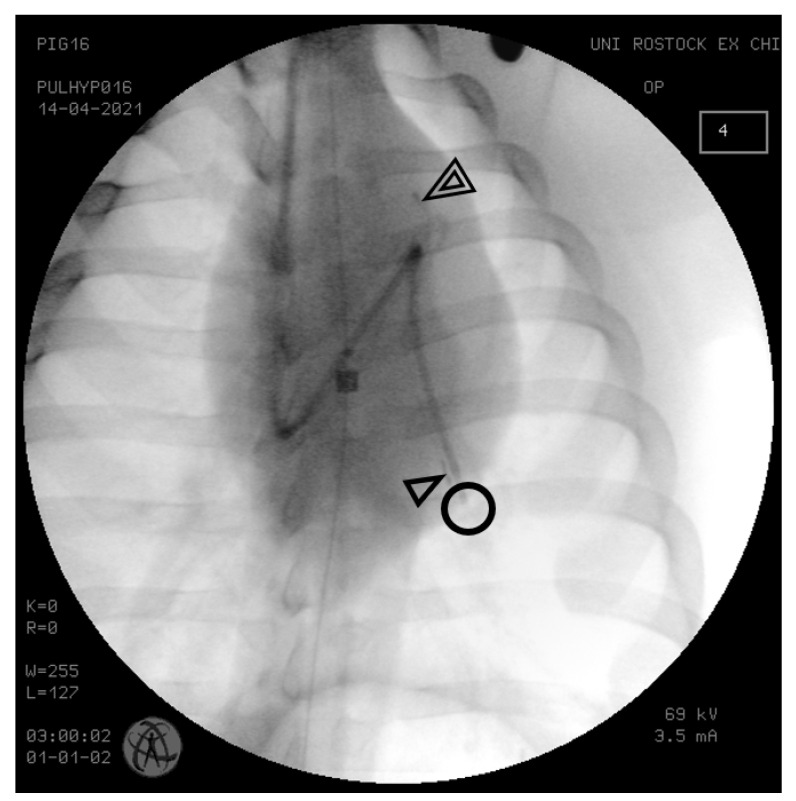
Thoracic X-ray to confirm correct positions of pulmonary artery catheters. A conventional pulmonary artery (Swan-Ganz) catheter was placed in the pulmonary artery for gold standard measurement of pulmonary artery pressure (circle). The tips of two Millar Mikro-Tip^TM^ pressure catheters were placed in a proximal (double open triangles) and in a distal branch of the pulmonary artery (open triangle).

For PAP measurement, a 7.5 Fr thermodilution pulmonary artery catheter was inserted into the proximal left pulmonary artery through an 8.5 Fr vascular sheath (both Arrow, Teleflex, Wane, PA, USA) placed in the right external jugular vein. Two 5 Fr Mikro-Tip™ Millar pressure catheters SPR-350 (Millar Instruments Inc., Houston, TX, USA) were surgically placed in the right internal jugular vein and advanced into the left pulmonary artery, the tip of the first one placed immediately behind the pulmonary valve (proximal) and the second one in a peripheral branch of the pulmonary artery (distal) (Figure 1). The distance between the sensors of both identical ikro-Tip^TM^ pressure catheters was approximated by measuring the outer distance of the connector of the pressure transducers, as reported previously [6,12]. The correct positions of all intravascular catheters were verified by posterior-anterior X-ray taken with the C-Arm Ziehm Vision (Ziehm Imaging, Nuremberg, Germany).

### 2.4. Data Acquisition and Processing

**Figure 2:**
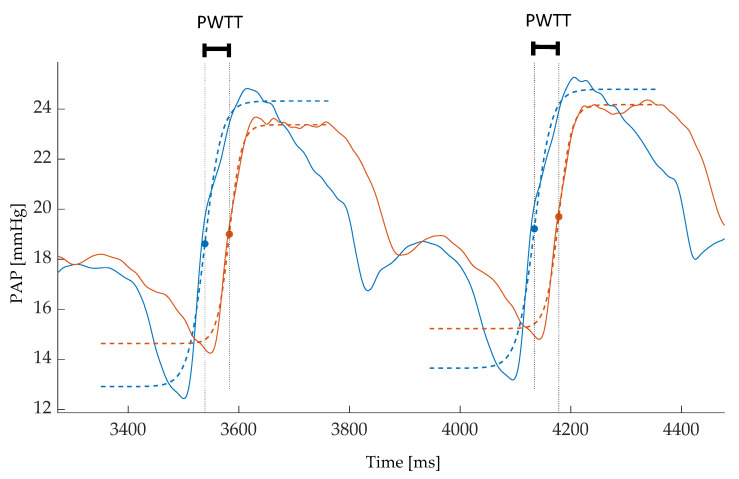
Pulse wave transit time (PWTT) between the proximal and distal pulmonary artery. With reference to the catheters shown in Figure 1 the proximal pressure curve is shown in blue, the distal one in red. Solid lines represent the respective pulmonary artery pressures in mmHg. Local pulse arrivals were determined by the inflection points of the fitted hyperbolic tangent (blue and red dotted lines) and marked by respective blue and red dots. PWTT was calculated as the time difference between the arrival times at the distal and the proximal measurement site (black dotted vertical lines).

Data were acquired at 10 kHz using bridge transducer amplifiers in combination with the respective hardware PowerLab 16/35 and software LabChart 8 (both ADInstruments, Dunedin, New Zealand). Data showing obvious mechanical noise caused by catheters bouncing within the pulmonary artery or against the pulmonary valve were excluded from the analysis. Additionally, data containing premature heart beats were also excluded. Stored data were exported from LabChart in a Matlab-compatible format (MATLAB^TM^ R2019b, The MathWorks, Inc., Natick, MA, USA). Applying the previously used intersecting tangent method for defining the arrival of the pulse waves did not result in sufficiently reproducible times. Hence, the precision of the pulse arrival measurement had to be improved. Therefore, a fitted hyperbolic tangent algorithm based on the work of Solà et al. was adapted for the current application and used for further analysis [13]. A hyperbolic tangent was fitted to the pressure curves. The inflection points of the hyperbolic tangent then defined the respective time of arrival of the pressure pulse at the proximal and distal catheters (filled dots in Figure 2). PWTT was then calculated as the time difference between the arrival of the pressure wave at the distal and the proximal site. Raw data and Matlab code used in this study are available upon request.

### 2.5. Statistics

Differences in end-inspiratory and end-expiratory PWTT and respective PAP were calculated and analyzed statistically using paired student’s t-tests of SigmaPlot 12.0 (Systat Software, Inc., San Jose, CA, USA). For comparison of standard deviations (std), an F-test was performed using MedCalc 19.3 (MedCalc Software Ltd., Oostende, Belgium).

## 3. Results

**Figure 3:**
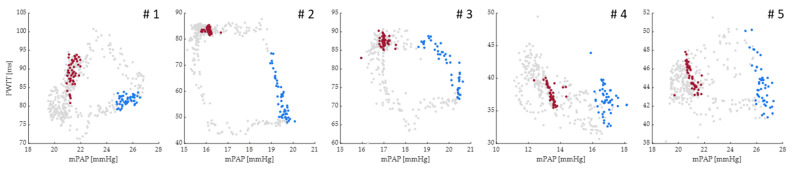
Scatter plot for the relation between mean pulmonary artery pressure (mPAP) and pulse wave transit time (PWTT) of each heartbeat during the respiratory cycle for all animals (numbered # 1–5 in the header of each cell) with each dot representing a heartbeat. The last heartbeat of inspiration is highlighted in blue while the last heartbeat of expiration is highlighted in red. All other heartbeats are shown in grey. Inspiratory PWTT is shorter and mPAP higher compared to expiration. The x and y coordinates of the colored datapoints were used in the statistical analysis presented in Table 2.

**Figure 4:**
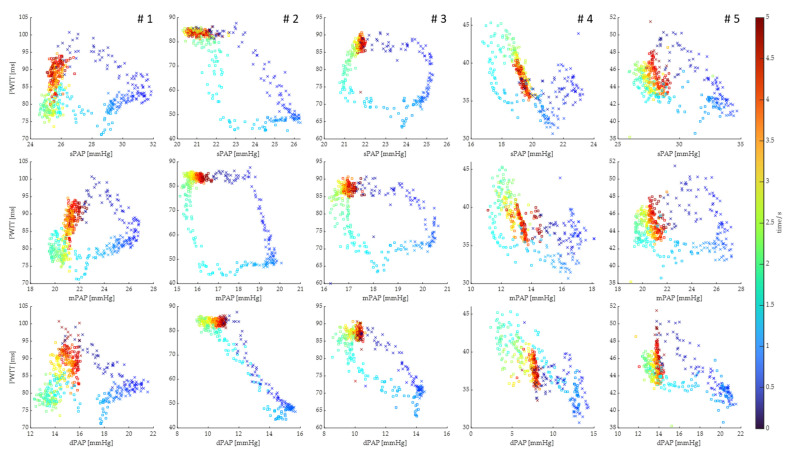
Scatter plots of systolic, mean and diastolic pulmonary artery pressure (sPAP, mPAP and dPAP) versus pulse wave transit time (PWTT) for each heartbeat during the respiratory cycle for all animals (numbered # 1–5 in the top row of each column). The respective time point of each heartbeat within the respiratory cycle is marked by a color gradient from blue (inspiration marked by colored crosses) to red (expiration marked by colored squares). The resulting hysteresis shows the dependency of PWTT on both PAP and the time point within the respiratory cycle.

The distance between the pressure sensors of the Mikro-Tip^TM^ catheters was 6.2–11.3 cm. For all 5 animals and for all heartbeats, the dynamic relationship between PWTT and mPAP is depicted during the breathing cycle (Figure 3 and Figure 4). Figure 3 shows the last heartbeats of inspiration in blue and the respective heartbeats of expiration in red with all other heartbeats indicated only faintly. The colored datapoints were used for the statistical analysis presented together with the respective *p*-values in Table 1 where the comparison revealed a significant increase in PAP and a significant drop in PWTT.

In order to analyze the time dependency of the changes in the relationship between PWTT and PAP in more detail, we visualized the respective time points of each heartbeat within the respiratory cycle by a color gradient in Figure 4. The resulting hysteresis shows a dependency of PWTT on both PAP and the timepoint within the respiratory cycle. At the beginning of inspiration, mPAP increased while PWTT initially remained constant, only to decrease rapidly when mPAP was about to reach its plateau. All animals showed a similar, almost rectangular, hysteresis when plotting PWTT and mPAP for the respective sequence of heartbeats within the respiratory cycle in a clockwise manner. This pattern could also be reproduced for sPAP and dPAP but with the latter being less pronounced.

Table 2 summarizes the mean values of PWTT, sPAP, mPAP and dPAP with the standard deviations of all pigs. When comparing the standard deviations of PWTT and of the different PAPs between periods of ventilation and of apnea, the standard deviations were significantly lower during apnea at *p* < 0.05 (Table 2).

## 4. Discussion

It was our aim to characterize the effect of ventilation on the relationship between PWTT and sPAP, mPAP, and also dPAP under resting conditions. We were able to demonstrate that PWTT within the pulmonary artery changed not only with PAP but also with the respiratory cycle for the first time. The shortening during inspiration was around 25 % of the average PWTT. This range of variability of PWTT for one value of PAP under resting conditions matched well with the one reported for acute pulmonary hypertension in our previous publication [6]. Interestingly, the inspiratory increase in PAP and the shortening of PWTT did not occur synchronously. PWTT became shorter at end-inspiration especially and became longer again towards the end of expiration, whereas the ventilation-induced changes in PAP seemed to be coupled more directly with the phases of the respiratory cycle (see Table 1).

Shortened PWTTs caused by artificially increased PAPs were demonstrated previously [6,14]. In fact, PWTT is known to become shorter when vessels are narrower, intravascular pressures are higher, vessel walls become stiffer and distances between proximal and distal pressure sensors become shorter [15]. Therefore, the fluctuation of PWTT for one corresponding PAP could be explained by several phenomena which are all related to the respiratory cycle.

Changes in arterial stiffness could affect PWTT independently of PAP. Lung filling is one determinant of pulmonary vascular resistance during mechanical ventilation, which is a function of a vessel’s diameter and its distensibility. As mentioned above, both are cofactors of the PWTT by itself [15,16]. The pulmonary vascular resistance (PVR) is minimal at normal resting volume or functional residual capacity (FRC), but increases as lung volume either moves towards total lung capacity (TLC) or decreases when exhaling towards residual volume (RV) [16,17]. PVR represents the cumulative resistances of the extra-alveolar vessels and the alveolar capillaries. Thus, with changing lung volumes both contribute to total PVR in opposite directions.

On the one hand, extra-alveolar vessels running through the lung parenchyma are mechanically attached to this tissue [18]. As the lung expands, their diameter increases due to radial traction on the vessel walls. As a result, the resistance of these large vessels decreases at large lung volumes. During lung collapse, however, the resistance of extra-alveolar vessels increases as their inherent elastic fiber contract [17].

On the other hand, the diameter of alveolar capillaries, including the capillaries and vessels in the corners of alveolar walls, behave in the opposite way. When lung volume approaches FRC alveoli are small and exert little external counterpressure on these small vessels. The result is increased vessel diameter and thus a lower PVR. When alveolar filling reaches TLC their resistance reaches its maximum [17,18].

In our experiment PEEP was set according to Talmor using an esophageal catheter to obtain a slightly positive transpulmonary pressure [11]. The individualized level of PEEP was intended to balance the collapsible forces within the mechanically ventilated lungs of animals under general anesthesia and muscle relaxation to obtain an end-expiratory lung volume close to FRC or slightly above. Therefore, inspiration could have increased global PVR and thereby shortened PWTT. Such inspiration-induced increases in PVR could explain—at least in part—the shortening of PWTT independently of PAP. Using an appropriate PEEP should also limit atelectasis in mechanically ventilated lungs, but cannot exclude them completely [19,20]. Since changes in PEEP were not performed during the investigation, changes in the PVR due to changing hypoxic pulmonary vasoconstriction could be excluded. Even those changes in PVR originating from hypoxic pulmonary vasoconstriction are not likely to occur during the tidal changes in lung volume [20].

The cyclic effects of ventilation on the relation of the three West zones to each other have not yet been described to affect the PWTT and could explain the inspiratory shortening of the PWTT in part, too. For clinical estimation of PAP and especially of pulmonary artery occlusion pressure (PAOP) three conditions must be met: A) PAOP should be assessed at end-expiration and B) the catheter should be placed in West zone III [18] and C) alveolar pressure should be lower than arterial and venous pressures ensuring that local perfusion between right and left ventricle is most undisturbed [19]. Therefore, it was our intention to place the pulmonary artery catheters in the lower lobe of the left lung (Figure 1). Inspiration increases the portion of the lung in conditions similar to West’s zones I and II. Thus, it is possible that the relative increase in these conditions during inspiration may have led to a more rapid propagation of the pulse wave and thus a shortening of the PWTT by occluding the distal end of the pulmonary artery tree. Thus, inspiration may have shortened the PWTT independently of the previously mentioned factors.

Since the PWTT is of particular interest as a surrogate parameter for the estimation of PAP, the ventilation-dependent changes in PWTT independent of PAP are of special concern.

During an apneic phase, the variations of PWTT and PAP were much smaller compared to phases with mechanical ventilation (see Table 2). Therefore, the respiratory state should be taken into consideration when using PWTT as a surrogate for PAP. For example, the PWV, easily measured by classical tomographic techniques, but not measured in this study due to large differences in the sensor distances, is used to estimate the current PAP. Laffon proposed an MR-based phase mapping in the main pulmonary artery for PWV calculation [21]. PWV correlated in a cohort of 15 patients with mPAP (r = 0.68). Comparing various MR-based techniques for mPAP calculation, Roeleveld could not confirm these findings [22]. All MR-based techniques involve an ECG-gating to extrapolate one representative cardiac cycle from around 20–40 image frames. Despite ECG-gating, the breathing status was not sufficiently addressed in these investigations and might have caused the relatively poor correlation coefficient reported in these studies.

Electrical impedance tomography (EIT), a non-invasive, radiation-free monitoring tool that allows real-time imaging of ventilation and pulmonary perfusion, has been proposed as an indirect means for estimating PAP [5]. Proença et al. first presented evidence for the usability of EIT-derived PAT, a combined parameter of the PWTT and the pre-ejection period, in norm- and hypoxic volunteers, to calculate PAP. Regardless of the respective respiratory state, ECG-gating of EIT signal was performed for 2 min and PAP was calculated [5]. In a consecutive investigation with 30 healthy volunteers, they reported a Pearson’s correlations coefficient of 0.76 for EIT-derived PAP estimates compared to echocardiographically measured PAP. However, again, the respiratory status remained unassessed [23].

### Limitations

Even though our study presents strong evidence for a shortening of PWTTs during inspiration, this experiment has obvious limitations. As we analyzed invasive data of multiple catheters in a porcine model, we had to perform our experiments in general anesthesia involving muscle relaxation. Therefore, it was impossible to evaluate normal spontaneous breathing conditions in which intrathoracic pressures become negative. Mechanical ventilation applies positive airway pressure during both inspiration and expiration. To assess the impact of such positive pressures on the cardiovascular system, we measured both airway and Peso simultaneously and used the resulting transpulmonary pressure to set PEEP [11].

The measurement of pulse wave arrival is challenging as the shape of the pressure curve changes considerably from the proximal to the distal pressure transducer. In our previous analyses, we used an intersecting tangent method [6]. Unfortunately, this method led to inconsistent results when trying to analyze the relatively small changes in PWTT under mechanical ventilation. Therefore, a more robust algorithm based on the hyperbolic tangent method needed to be developed. This is why the results of the current study cannot be compared directly with those of our previous one.

Since the spontaneous cardiac rhythm was not synchronized with that of the respiratory cycle some heartbeats occurred during both inspiration and expiration. To address this issue, we only used the last complete heartbeats of the inspiration and of the expiration phase for our statistical analysis in Table 2.

While airway pressures were measured with a catheter placed directly in the airway and recorded in the data acquisition system, we were unable to export any data from the ventilator. Therefore, no tidal volumes, which would have given useful insights into the filling status of the lungs, were available. This latter limitation will be resolved in upcoming experiments.

In addition, we assume the complex interaction between heart and lungs to also have effects on the heart rate, vascular tone, stroke volume and cardiac output, which influence the measured PWTT and its relation to PAP. However, we have no data to quantify such heart-lung interaction [24,25].

## 5. Conclusions

Non-synchronous ventilation-induced changes in PWTT and PAP were found. Inspiration caused a significant shortening of PWTT. Therefore, it is suggested that the respiratory cycle should be considered when using PWTT as a surrogate for PAP. These findings provide novel insights into the mechanisms of heart-lung interaction which warrant further explorations.

## Figures and Tables

**Table 1:** Means and standard deviations of pulse wave transit times (PWTT) and systolic, mean, and diastolic pulmonary artery pressures (sPAP, mPAP, dPAP) together with respective *p*-values for all animals during pressure-controlled mechanical ventilation for inspiration and expiration. In all pigs, PWTT dropped significantly (*) during inspiration, while sPAP, mPAP and dPAP increased significantly (*) at *p* ≤ 0.05.

	PWTT [ms]			sPAP [mmHg]			mPAP [mmHg]			dPAP [mmHg]		
	Expiration	Inspiration	*p*-Value	Expiration	Inspiration	*p*-Value	Expiration	Inspiration	*p*-Value	Expiration	Inspiration	*p*-Value
1	88.9 ± 3.4	81.8 ± 1.6 *	0.001	25.7 ± 0.4	30.4 ± 0.9 *	0.001	21.4 ± 0.3	25.5 ± 0.8 *	0.001	15.4 ± 0.4	20.2 ± 1.0 *	0.004
2	81.1 ± 54	57.8 ± 8.4 *	0.001	21.1 ± 0.3	25.3 ± 0.9 *	0.001	16.1 ± 0.1	19.4 ± 0.6 *	0.001	10.7 ± 0.2	13.8 ± 1.1 *	0.001
3	87.5 ± 1.2	83.5 ± 0.9 *	0.001	21.8 ± 0.1	24.8 ± 0.4 *	0.001	17.0 ± 0.2	19.9 ± 0.6 *	0.001	10.3 ± 0.1	12.3 ± 1.3 *	0.001
4	37.7 ± 1.3	36.7 ± 2.1 *	0.001	19.3 ± 0.3	22.3 ± 0.5 *	0.001	13.4 ± 0.3	16.8 ± 0.4 *	0.001	7.7 ± 0.4	11.2 ± 1.9 *	0.001
5	45.2 ± 1.3	44.3 ± 2.3 *	0.001	28.2 ± 0.5	32.7 ± 1.2 *	0.001	21.0 ± 0.4	26.4 ± 0.5 *	0.001	13.9 ± 0.2	18.5 ± 1.6 *	0.001

**Table 2:** Means and standard deviations of pulse wave transit times (PWTT), systolic, mean, and diastolic pulmonary artery pressures (sPAP, mPAP, dPAP) together with respective *p*-values for all animals during pressure-controlled mechanical ventilation and during an expiratory hold maneuver, referred to as apnea. In all animals, the standard deviations of all parameters were significantly (*) lower during apnea at *p* ≤ 0.05 (F-test for differences in standard deviations).

	PWTT [ms]			sPAP [mmHg]			mPAP [mmHg]			dPAP [mmHg]		
	Ventilation	Apnoe	*p*-Value	Ventilation	Apnoe	*p*-Value	Ventilation	Apnoe	*p*-Value	Ventilation	Apnoe	*p*-Value
1	84.6 ± 5.8	71.3 ± 1.4 *	0.001	26.9 ± 2.1	29.7 ± 0.6 *	0.001	22.2 ± 2.0	23.8 ± 0.2 *	0.001	15.9 ± 2.3	16.2 ± 0.4 *	0.001
2	73.3 ± 14.7	80.9 ± 0.8 *	0.001	22.4 ± 1.8	21.3 ± 0.5 *	0.001	16.9 ± 1.5	16.9 ± 0.2 *	0.001	11.7 ± 1.9	11.5 ± 0.4 *	0.001
3	82.2 ± 7.1	62.5 ± 1.2 *	0.001	22.5 ± 1.4	24.4 ± 0.2 *	0.001	17.7 ± 1.3	18.9 ± 0.3 *	0.001	11.0 ± 1.7	12.5 ± 0.4 *	0.001
4	37.8 ± 2.9	51.8 ± 0.7 *	0.001	19.7 ± 1.4	19.0 ± 0.4 *	0.001	13.9 ± 1.7	13.1 ± 0.3 *	0.001	8.2 ± 2.9	7.9 ± 0.3 *	0.001
5	44.6 ± 2.0	46.4 ± 1.1 *	0.001	29.1 ± 2.3	27.6 ± 0.5 *	0.001	22.0 ± 2.3	20.7 ± 0.4 *	0.001	15.4 ± 2.7	13.5 ± 0.4 *	0.001

## Data Availability

The data presented and Matlab code in this study are available on request from the corresponding author. The data are not publicly available due to copyright issues.
